# An audit of influenza and pneumococcal vaccination in rheumatology outpatients

**DOI:** 10.1186/1471-2474-8-58

**Published:** 2007-07-04

**Authors:** Evin Sowden, William S Mitchell

**Affiliations:** 1 Department of Rheumatology, Furness General Hospital, Barrow-in-Furness, UK

## Abstract

**Background:**

Influenza and pneumococcal vaccination are recommended for a number of clinical risk groups including patients treated with major immunosuppressant disease modifying anti-rheumatic drugs. Such immunisation is not only safe but immunogenic in patients with rheumatic diseases. We sought to establish dual vaccination rates and significant influencing factors amongst our hospital rheumatology outpatients.

**Method:**

We audited a sample of 101 patients attending hospital rheumatology outpatient clinics on any form of disease modifying treatment by clinical questionnaire and medical record perusal. Further data were collected from the local immunisation coordinating agency and analysed by logistic regression modelling.

**Results:**

Although there was a high rate of awareness with regard to immunisation, fewer patients on major immunosuppressants were vaccinated than patients with additional clinical risk factors against influenza (53% vs 93%, p < 0.001) or streptococcus pneumoniae (28% vs 64%, p = 0.001). The presence of additional risk factors was confirmed as significant in determining vaccination status by logistic regression for both influenza (OR 10.89, p < 0.001) and streptococcus pneumoniae (OR 4.55, p = 0.002). The diagnosis of rheumatoid arthritis was also found to be a significant factor for pneumococcal vaccination (OR 5.1, p = 0.002). There was a negative trend suggesting that patients on major immunosuppressants are less likely to be immunised against pneumococcal antigen (OR 0.35, p = 0.067).

**Conclusion:**

Influenza and pneumococcal immunisation is suboptimal amongst patients on current immunosuppressant treatments attending rheumatology outpatient clinics. Raising awareness amongst patients may not be sufficient to improve vaccination rates and alternative strategies such as obligatory pneumococcal vaccination prior to treatment initiation and primary care provider education need to be explored.

## Background

Influenza infection is responsible for considerable morbidity and mortality in epidemic years [1], whilst streptococcus pneumoniae infection accounts for 48% of bacteriologically confirmed community acquired pneumonias [2]. A number of groups have been identified as being at high risk for whom vaccination is currently recommended in the United Kingdom (UK), including individuals over 65 years of age or with heart disease, chronic lung disease, diabetes, chronic renal failure, chronic liver failure or hyposplenism [3]. Patients with inflammatory rheumatic diseases have an increased incidence of infections [4] including those of the respiratory tract [5]. Treatment with corticosteroids and some other Disease Modifying Anti-Rheumatic Drugs (DMARDs) has been implicated as contributing to the increased susceptibility to infection [5,6]. More recently, this has also been highlighted for biologic therapies targeting tumour necrosis alpha inflammatory pathways [7]. Both influenza and pneumococcal vaccination are safe and immunogenic in patients with rheumatic diseases who are taking DMARDs [8-11]. National UK guidelines specifically recommend immunisation for patients on systemic steroids at a dose of 20 mg or more [3]. It is our unit's current policy to provide patients started on other DMARDs with written information (Arthritis Research Campaign, UK) advising influenza and pneumococcal immunisation for major immunosuppressants in line with national guidance [12] although we neither provide a vaccination service nor withhold major immunosuppressant DMARD treatment until the patient is vaccinated. Influenza vaccine uptake has previously been noted to be as low as 56% amongst rheumatoid arthritis patients on major immunosuppressant therapies [13] whilst uptake amongst the over 65 year age group in the year 2005–6 was 75% for influenza and 64% for pneumococcal vaccine in the UK [14] prompting us to audit immunisation rates for both influenza and pneumococcal vaccine amongst all patients attending outpatient rheumatology clinics at our district general hospital and explore associated influencing factors.

## Methods

Patients were eligible for inclusion if they were on DMARD treatments including systemic corticosteroids (over 20 mg per day for at least 1 month in the last 12 months) and biologic therapy whilst attending a doctor or nurse-led adult rheumatology outpatient clinic at our district general hospital. No exclusion criteria were specified. A single page questionnaire gathering information on general practitioner, age, gender, rheumatological diagnosis, significant comorbidities, current treatment, vaccination awareness, vaccination status (influenza vaccine in the previous 12 months and pneumococcal vaccine in the previous 5 years) and underlying motive for vaccine refusal if applicable was created to standardise data collection amongst auditors. A large practice was defined as a general practice at which more than 6 audit patients were registered. Whilst the questionnaire was not formally validated prior to the study, its design was discussed with our clinical audit department and then reviewed in the initial stages of the project. No questionnaire modifications were deemed necessary and the audit was continued as initially planned. Our patient recruitment strategy was designed to maximise the number of audited patients over a short period of time. To this end, questionnaires were simultaneously distributed to two nurse practitioners, two specialist registrars and one consultant in our rheumatology department in September 2006. Individual auditors then consecutively screened and selected for audit suitable patients attending their respective outpatient clinics until a predefined target of 100 audited patients was achieved in October 2006. Data were collected from patient interview and available hospital records by individual auditors at the time of clinical review. There were no recorded instances of refusal to participate in our audit by a patient. Additional corroborative information was sought from the immunisation coordinating Lancashire and South Cumbria Agency, Preston, UK. This was released to our clinical audit department after formal approval by our appointed Caldicott guardian in charge of patient data protection, merged with interview data and then released in anonymised format to the investigators. Formal approval to carry out this audit was sought and granted by our clinical audit department. The Statistical analysis was performed using software supplied by Minitab Inc, USA and Creostat HB, Sweden.

## Results

The main characteristics of our sample of 101 patients are summarised in table 1. The age range was 32 to 87 years with a mean age of 60.6 years. There was a predominance of female patients and patients attending large general practices. A rheumatological diagnosis was available for 99 patients and 4 of these had more than one diagnosis. The main diagnostic group consisted of rheumatoid arthritis, with psoriatic arthritis and polymyalgia rheumatica (PMR) or giant cell arteritis (GCA) representing two-thirds of the remaining diagnoses. In total 56 patients had additional risk factors (other than major immunosuppressant DMARDs) for which influenza or pneumococcal immunisation are usually indicated. There were 33 patients over 65 years of age thus making age the most frequent of these risk factors and accounting for 59% of patients in this group. Methotrexate was the most commonly encountered DMARD drug in 48 patients and 27 patients were on a combination of two or more agents. We identified 76 patients on major immunosuppressant DMARDs (methotrexate, leflunomide, cyclosporine, azathioprine, biologics and corticosteroids) for whom immunisation is considered to be indicated and 25 patients on minor immunosuppressant DMARDs (sulphasalazine, penicillamine, hydroxychloroquine and gold) for whom immunisation is not considered indicated.

**Table 1:** Main characteristics of sample population

*Demographics*	
Total number of patients	101
Age range in years (mean)	32–87 (60.6)
Female	70
Registered to Large Practice	54

*Diagnosis*	

Rheumatoid Arthritis	71
Psoriatic Arthritis	11
PMR or GCA	9
SLE	6
Other	6

*Risk factors*	

>65 years	33
Chronic lung disease	16
Ischaemic heart disease	12
Diabetes	11
Any risk factor	56

*DMARD therapy*	

Immunosuppressant^1^	76
Non-immunosuppressant^2^	25

^1^Methotrexate, Leflunomide, Cyclosporine, Azathioprine, Biologic, Steroid

^2 ^Sulphasalazine, Penicillamine, Hydroxychloroquine, Gold

We compared the vaccination profile of patients with and without additional risk factors according to their use of major immunosuppressant DMARDs as shown in Table 2. Amongst our audit sample 45 patients had no additional risk factors amongst which 32 were on major immunosuppressant DMARDs. Awareness of individual need for immunisation was generally high, even in patients with no additional risk factors and on minor immunosuppressant DMARDs. Nevertheless, fewer patients on major immunosuppressant DMARDs were offered immunisations than patients with additional risk factors (p < 0.001) as evidenced by the influenza and pneumococcal immunisation rates with 93% vs 53% (p < 0.001) and 64% vs 28% (p = 0.001) of patients being vaccinated against influenza and streptococcus pneumoniae respectively. Interestingly, the proportion of influenza vaccinated patients on major immunosuppressant DMARDs without additional risk factors is not significantly different from that of patients on minor immunosuppressant DMARDs (53% vs 54%, p = 0.965), as was also the case for pneumococcal vaccine (28% vs 38%, p = 0.497). Amongst patients offered influenza or pneumococcal immunisation by their general practitioners, overall uptake of influenza or pneumococcal vaccine was 90%, with only 4 patients refusing their influenza or pneumococcal vaccination offers. Stated reasons for refusal included vaccine allergy, patient belief that vaccination was unnecessary or that vaccination was contraindicated.

**Table 2:** Vaccination profile of sample population (%)

	Total	Awareness	GP offer	Influenza immunisation	Pneumococcal immunisation
No risk factor + Minor immunosuppressant	13	10 (77)	8 (62)	7(54)	5(38)
No risk factor + Major Immunosuppressant	32	26 (81)	17 (53)	17(53)	9(28)
Risk factor + Any Immunosuppressant	56	55 (98)	53 (95)	52(93)	36(64)
Chi-square test of association	-	-	p < 0.001	p < 0.001	p = 0.003

We investigated the factors influencing immunisation amongst our sample of patients by logistic regression modelling using presence of additional risk factor, size of general practice, diagnosis of rheumatoid arthritis, major immunosuppressant drug therapy and gender as predictor variables. Results are displayed in tables 3 and 4.

**Table 3:** Factors influencing influenza vaccination

	Odds Ratio	95% CI	P value
Risk factor	10.89*	3.24–36.65	<0.001
Large Practice	2.78	0.92–8.36	0.069
Rheumatoid Arthritis	1.50	0.48–4.69	0.489
Female	0.90	0.25–3.26	0.871
Major Immunosuppressant^#^	0.63	0.18–2.20	0.472

^#^Methotrexate, Leflunomide, Cyclosporine, Azathioprine, Biologic, Steroid

* Significant result (p < 0.05)

**Table 4:** Factors influencing pneumococcal vaccination

	Odds Ratio	95% CI	P value
Rheumatoid arthritis	5.10*	1.78–14.59	0.002
Risk factor	4.53*	1.76–11.62	0.002
Large Practice	0.80	0.31–2.06	0.646
Female	0.75	0.27–2.10	0.583
Major Immunosuppressant^#^	0.35	0.11–1.07	0.067

^#^Methotrexate, Leflunomide, Cyclosporine, Azathioprine, Biologic, Steroid

* Significant result (p < 0.05)

In the case of influenza vaccination the presence of an additional risk factor (OR 10.89, 95% CI 3.24–36.65) was the only significant factor influencing the odds ratio for immunisation, whilst a trend was apparent for large practices (OR 2.78, 95% CI 0.92–8.36). There was no detectable effect of major immunosuppressant use.

Significant factors influencing pneumococcal vaccination included a diagnosis of rheumatoid arthritis (OR 5.10, 95% CI 1.78–14.59) and the presence of additional risk factors (OR 4.53, 95% CI 1.76–11.62). There was a notable negative trend for the odds ratio with major immunosuppressant use (OR 0.35, 95% CI 0.11–1.07).

## Discussion

Given the increased susceptibility to infection amongst patients with inflammatory rheumatic diseases and the potential to counteract this risk with influenza and pneumococcal vaccination, it is important to ascertain the effect that current education and immunisation practice are having on rheumatology patients.

Our audit has shown a high rate of immunisation awareness amongst our patients as could be expected from our unit's policy of routinely counselling patients starting disease modifying treatments and providing written drug information leaflets. A small number of patients on minor immunosuppressant DMARD therapy and without additional risk factors requiring immunisation believed they needed vaccinating and this was reflected in their rate of vaccination offer and uptake. Information on risk factors was collected by direct patient questioning with access to clinical records during patient consultation, and it would seem unlikely that any major risk factors would have been missed in the audit process thus raising the possibility that the perceived immunosuppressant effects of rheumatic illness or the perceived risk of minor immunosuppressant drug therapy may be affecting patients' and their family physicians' immunisation strategies. The first possibility is supported by finding rheumatoid arthritis to be a significant factor influencing pneumococcal vaccination rates through our logistic regression model. A number of our patients will inevitably eventually turn to major immunosuppressant DMARDs as rescue therapies and we are therefore not concerned by what appears to be over-vaccination on the part of our general practitioner colleagues.

Patients who are on major immunosuppressants are nevertheless less commonly offered vaccination or vaccinated as compared to patients with other risk factors despite the high levels of patient awareness and uptake suggesting that a proportion of our high risk patients are not actively pursuing known immunisation advice nor being correctly identified by their family physicians. Notwithstanding our small audit sample size, there is even a trend suggesting that major immunosuppressant use may be an independent negating factor in the setting of pneumococcal vaccination. Interestingly, patients belonging to larger general practices with higher numbers of rheumatology patients are likely to achieve better coverage for influenza immunisation and this may suggest that organisational factors in individual practices may play an important role here.

As influenza vaccination is seasonal, it would be impractical and difficult given our limited resources to offer annual immunisation directly in our rheumatology outpatient clinics when there are funded agencies dedicated to this purpose operating within our area. There is also a concern that patients could unnecessarily be vaccinated more than once by different providers and for these reasons we maintain that responsibility for immunisation should continue to rest with primary care providers. As immunosuppressant therapy is mostly initiated in rheumatology outpatient clinics, one possible approach to improve current practice and perhaps immunisation efficacy would be to routinely request patient vaccination prior to rather than after the initiation of major immunosuppressant therapy as we do at present. Whilst feasible for pneumococcal vaccine, this approach may be limited by the availability of influenza vaccine outwith the immunisation calendar. We would therefore also recommend contacting primary health care providers before the start of each annual immunisation season to raise general awareness about the need to vaccinate any patient on major immunosuppressant therapy in order improve targeting of existing rheumatology patients in the community.

## Conclusion

Rheumatology outpatients are not being appropriately targeted by the influenza and pneumococcal vaccination efforts on the part of primary care physicians. Raising patient awareness is not sufficient to ensure optimal immunisation thus calling for alternative strategies on the part of rheumatology services to improve vaccination coverage

## Competing interests

The author(s) declare that they have no competing interests.

## Authors' contributions

ES proposed, designed, performed statistical analysis and wrote the first draft report of the audit study. WM approved, contributed to the design, took part in the data collection and edited the first draft report. All authors read and approved the final manuscript.

## Pre-publication history

The pre-publication history for this paper can be accessed here:


